# Successful endoscopic sclerotherapy for anastomotic tumor hemorrhage: A case report

**DOI:** 10.1002/deo2.262

**Published:** 2023-06-19

**Authors:** Jinfeng Ren, Tianyu Liu, Yunzhi Zhang, Ying Bi, Zhengying Yang, Xihui Yuan

**Affiliations:** ^1^ Unit 4 of Gastroenterology Center Suining Central Hospital Suining Sichuan China

**Keywords:** anastomotic tumor, endoscopic sclerotherapy, hemostasis, lauromacrogol, tumor bleeding

## Abstract

A 71‐year‐old male developed anastomotic tumor bleeding after subtotal gastrectomy (Billroth II), and the surgery was refused due to coronary atherosclerotic heart disease. Endoscopic sclerotherapy (ES) in the treatment of esophagogastric variceal bleeding has been commonly reported, but few in non‐variceal upper gastrointestinal bleeding. As far as we know, endoscopic sclerotherapy for malignant tumor hemorrhage has not been reported. Here a valuable case is presented: endoscopic sclerotherapy was performed on the anastomotic tumor bleeding, which is an effective try in a particular period.

## INTRODUCTION

Upper gastrointestinal bleeding remains a commonly encountered disease that seriously endangers lives. Endoscopic treatment is considered to have surpassed surgery as the treatment of choice for most upper gastrointestinal bleeding with the advantages of being timely, accurately, and intuitively.^1^ In recent years, endoscopic sclerotherapy (ES) is widely used in gastroesophageal varices bleeding.^2^ The main mechanism is thrombosis caused by vascular endothelial injury, thereby blocking blood vessels. At present, the common hemostasis methods for non‐variceal gastrointestinal bleeding mainly include drug spraying or injection, argon ion coagulation, metal titanium clipping, and so forth.^3^, ^4^ A few studies certified that ES could improve the immediate hemostasis ratio and reduce the rebleeding ratio in the treatment of peptic ulcer^5^ but it is not used in the treatment of malignant tumor hemorrhage so far. Here we report a case of successful ES for anastomotic tumor hemorrhage after subtotal gastrectomy.

## CASE REPORT

A 71‐year‐old male presented with recurrent melena for more than nine months caused by anastomotic tumor bleeding diagnosed by gastric endoscopy. Nine months ago, the patient presented melena, accompanied by dizziness and fatigue. Gastroscopy showed an anastomotic mass with a surface ulcer, post‐gastrectomy (Billroth II), and residual gastritis. Biopsy and pathological examination of the anastomotic mass confirmed it poorly differentiated adenocarcinoma. In the past 9 months, only blood transfusion therapy was accepted, and the surgery was repeatedly refused because the patient had coronary atherosclerotic heart disease. This patient underwent subtotal gastrectomy (Billroth II) for a duodenal ulcer more than 20 years ago. Physical examination showed an anemic face, pale skin, and mucous membrane. The laboratory tests were as follows: erythrocyte count 3.67×10^12^/L, hemoglobin 63g/L, and platelets 399×10^9^/L. Fecal occult blood test was positive. And there is no obvious abnormality in coagulation function and other laboratory tests. Anastomotic carcinoma bleeding after subtotal gastrectomy was diagnosed again, and post‐gastrectomy, chronic hemorrhagic anemia, and moderate anemia were complicated. Surgery was denied as before, whereas a positive decision, endoscopy hemostasis was finally accepted and consented.

**FIGURE 1:**
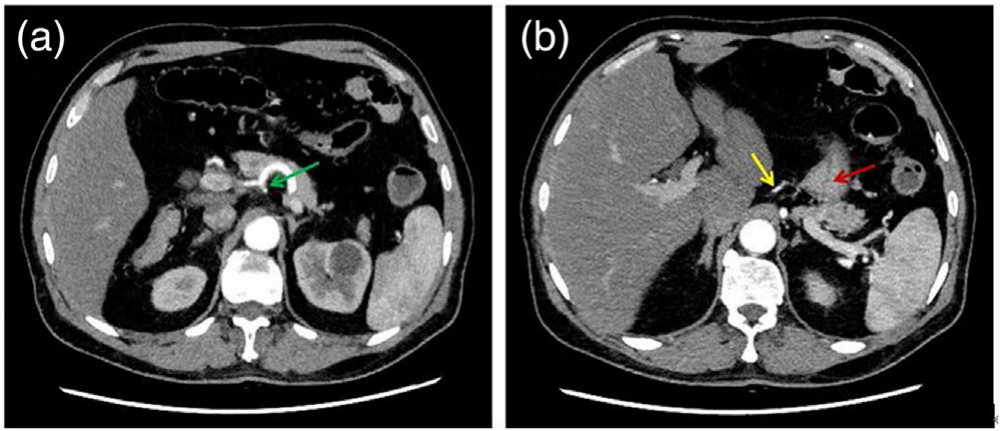
Abdominal enhanced computed tomography scan. (a) The left gastric artery from the celiac trunk (arrow). (b) The nutrient artery of the tumor from the left gastric artery (yellow arrow) and the residual stomach (red arrow).

**FIGURE 2(a):**
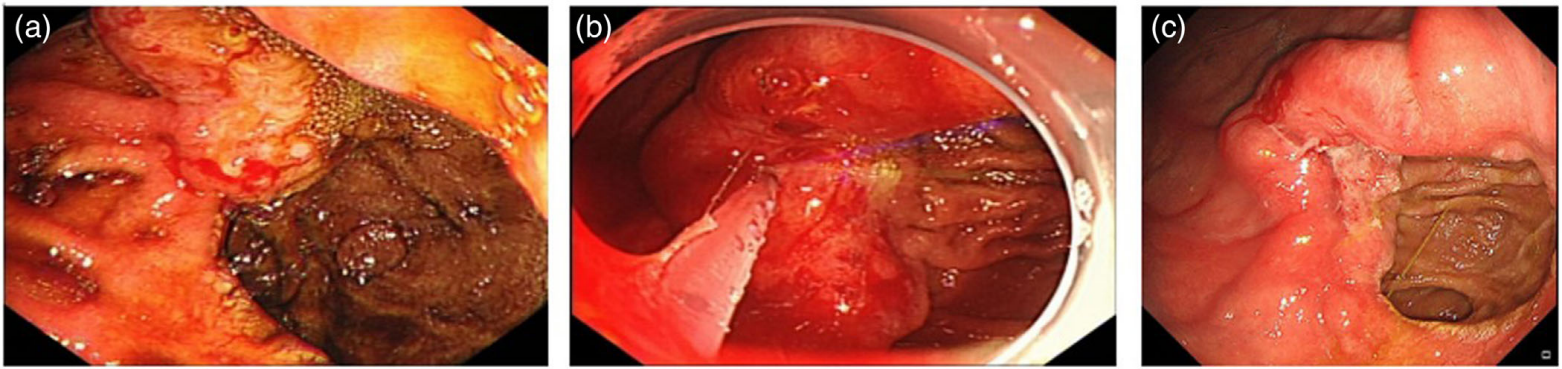
Anastomotic carcinoma and active hemorrhage before treatment. (b) Endoscopic sclerotherapy and tissue glue injection for anastomotic carcinoma bleeding. (c) Endoscopic review shows the lesion hardened and the size of the carcinoma looks decreased.

Previous abdominal enhanced CT scan suggested that the nourishing artery of the tumor (Figure 1a) originated from the left gastric artery (Figure 1b) emanating from the celiac trunk. During the endoscopic operation (Video 1), gastroscopy (GIF‐H290, OLYMPUS) showed that the mucosa of the lesion tissue was congested and swollen, and with an ulcer on the surface. Bloodstains were observed on the lesion surface and in the gastric cavity (Figure 2a). Lauromacrogol (Tianyu Pharmaceutical, Xi'an, China) was first injected into the submucosa with each point about 0.2 ml, with a total amount of about 2 ml. The lesion tissue gradually became white, oozing of blood decreased. The nourishing artery of the carcinoma appeared jet bleeding after injecting into the stump of the blood vessel, and then the puncture needle (VDK‐IN‐23‐180‐2304‐A; Jiangsu Vedkang Medical Science and Technology Co., Ltd) was inserted into the tissue rupture, the blood return was seen in the needle sheath (Video 1, Arrow), and 1 ml of tissue glue (G‐NB‐2 [1 ml], GEM S.R.L Ge) was subsequently injected into blood vessel immediately and precisely to block it (Figure 2b). The lesion became hardened, and the bleeding stopped immediately. Finally, porcine fibrin adhesive (5ml, Guangzhou Bioseal Biotech Co., Ltd) was sprayed on the surface of the lesion to protect the wound after treatment. Two days later, the hemoglobin raised to 82g/L, and the fecal occult blood test was negative.

The gastroscopy review after one month showed the lesion hardened, and the size of the carcinoma looks decreased (Figure 2c). There was no active hemorrhage and the hemoglobin raised to 154g/L.

## DISCUSSION

At present, ES is mainly used in patients with esophageal variceal bleeding. And thrombosis caused by vascular endothelial injury is a recognized hemostatic mechanism. In arterial hemorrhagic diseases such as ulcer bleeding, this mechanism has not been elucidated. In this case, ES is used for the treatment of anastomotic malignant tumor bleeding, which has no similar report so far, simple operation and minimal invasion are the advantages compared with surgical resection.^6^ In this treatment, Lauromacrogol was injected into the submucosa to block submucosal blood flow. And then the nourishing artery of the carcinoma appeared spurting bleeding, the tissue glue was injected into the blood vessel precisely when the puncture needle was inserted into the tissue rupture and the blood return was seen in the needle sheath. Therefore, it confirmed that the tissue glue injection is an intravascular injection, and it would not cause massive tissue necrosis and rebleeding after the tissue glue was injected accurately into the nourishing artery. A gastroscopy review showed there was no active hemorrhage, and the size of the carcinoma looks decreased after one month. The causes of tumor shrinkage may be related to the following reasons: Lauromacrogol can directly damage vascular endothelium, promote thrombosis, and further cause tissue fibrosis, thus causing vascular occlusion and tumor atrophy.^6^ Due to the rapid coagulation of tissue glue, the bleeding nutrient artery was immediately blocked. The submucosal blood supply was blocked by submucosal injection, resulting in necrosis and shedding of the tumor. In terms of intraoperative safety, since the blood flow was to the rupture and the amount of tissue glue was only 1ml, it would not cause ectopic embolization of tissue glue. And the Lauromacrogol was injected into the submucosa, not directly into the blood vessel, so there was no risk of ectopic embolism. Besides only 0.2 ml of lauromacrogol was injected at each point, which would not cause a large area of ulceration or perforation. Therefore, the treatment is very safe and reliable. At present, there are many treatments for anastomotic tumor hemorrhage, such as argon plasma coagulation, high‐frequency soft coagulation, interventional radiology, and so forth. We have tried to use argon plasma coagulation or high‐frequency soft coagulation for peptic ulcer hemostasis in the past work, but the effect for arterial bleeding is not very satisfactory, and it is easy to cause new vascular injury or ulcer base damage to aggravate bleeding. And interventional embolization is also one of the treatment methods for tumors. In this case, interventional embolization was not first chosen due to the consideration of the lack of abundant anastomotic vessels, which may cause potential complications such as ischemic necrosis of anastomotic tissue or anastomotic fistula after embolization. If the patient suffered massive bleeding again during the follow‐up, we would also consider the treatment of vascular interventional embolization.

To sum up, ES was performed on the anastomotic tumor bleeding by us, and the hemostasis and tumor progression appeared to be controlled. The long‐term treatment effect needs to be further observed.

## CONFLICT OF INTEREST STATEMENT

None

## Supporting information

Video

## References

[1] Feinman M , Haut ER . Upper gastrointestinal bleeding. Surg Clin North Am 2014; 94: 43–53. 24267496 10.1016/j.suc.2013.10.004

[2] Al‐Khazraji A , Curry MP . The current knowledge about the therapeutic use of endoscopic sclerotherapy and endoscopic tissue adhesives in variceal bleeding. Exp Rev Gastroenterol Hepatol 2019; 13: 893–7. 10.1080/17474124.2019.1652092 31389265

[3] Laine L , Barkun AN , Saltzman JR , Martel M , Leontiadis GI . ACG clinical guideline: Upper gastrointestinal and ulcer bleeding. Off J Am Coll Gastroenterol 2021; 116: 899–917. 10.14309/ajg.0000000000001245 33929377

[4] Szura M , Pasternak A . Upper non‐variceal gastrointestinal bleeding – Review the effectiveness of endoscopic hemostasis methods. World J Gastrointest Endosc 2015; 7: 1088–95. 26421105 10.4253/wjge.v7.i13.1088 PMC4580950

[5] Ertem S , Kasirga E , Sarul AR , Altinay ZA . The role of endoscopic injection sclerotherapy in upper gastrointestinal bleeding due to peptic ulcers. Turk Soc Gastroenterol 1999; 10: 15–7.

[6] Qu H , Lei X , Hu L *et al*. Successful endoscopic sclerotherapy using lauromacrogol injection for laryngopharyngeal hemangioma. Ear Nose Throat J 2021; 100: 662–6. 34551625 10.1177/01455613211043690

